# The transmission of bovine leukemia virus to calves occurs mostly through colostrum and milk

**DOI:** 10.14202/vetworld.2024.2918-2924

**Published:** 2024-12-26

**Authors:** Daniel Lazzari Quadros, Kalinka Puhl, Vitoria Agnoletto Ribeiro, Rafael Frandoloso, Luiz Carlos Kreutz

**Affiliations:** 1 Graduate Program in Bioexperimentation, Laboratory of Advanced Microbiology and Immunology, School of Agricultural Sciences, Innovation and Business. University of Passo Fundo, 99052-900 Passo Fundo, RS, Brazil; 2 School of Veterinary Medicine. Atitus Education. 99070-220 Passo Fundo, Brazil

**Keywords:** diagnosis, epidemiology, retrovirus, vertical transmission

## Abstract

**Background and Aim:**

Enzootic bovine leukemia is highly prevalent in most dairy farms, and strategies to reduce both vertical and horizontal transmission are being investigated. In this study, we aimed to investigate the rate of *in utero* infection, transmission of bovine leukemia virus (BLV) to calves through colostrum and milk, and the effectiveness of colostrum and milk pasteurization in reducing BLV transmission to calves.

**Materials and Methods:**

This study included four groups of calves from seropositive and seronegative cows. Group 1: Calves from BLV-positive cows (n = 11) were fed pasteurized colostrum and milk; Group 2: Calves from BLV-negative cows (n = 9) were fed pasteurized colostrum and milk; Group 3: calves from BLV-positive cows (n = 16) were fed unpasteurized colostrum and milk; and Group 4: calves from BLV-negative cows (n = 9) were fed unpasteurized colostrum and milk. *In utero* infection was evaluated using blood samples collected from calves before colostrum ingestion (day 0), and BLV transmission through colostrum and/or milk was evaluated by collecting blood samples after colostrum ingestion (days 1, 7, and 30). Samples seropositive on days 0 and/or 30 were also analyzed for the presence of viral DNA by nested polymerase chain reaction (nPCR).

**Results:**

All calves born to BLV seronegative cows (Groups 2 and 4) tested negative on days 0 and 30, indicating a lack of virus transmission via tank milk. Among the calves from Group 1, we found one *in utero* infection, and among the nine calves serologically positive on day 30, we found four positives by nPCR. Within Group 3, we found one *in utero* infection, and among the 10 calves serologically positive on day 30, we found 7 also positive by nPCR.

**Conclusion:**

The transmission of BLV through colostrum is central to the persistence of this virus in dairy cattle. Molecular detection of BLV in seropositive calves during the 1^st^ month of life, followed by culling, may be a valuable eradication strategy.

## Introduction

Bovine leukemia virus (BLV) is an oncogenic retrovirus that causes enzootic bovine leukemia (EBL), a tumoral disease that is most frequently observed in dairy cows [1]. The infection is usually asymptomatic; however, in some cows, the virus may cause persistent lymphocytosis (PL) because of the nonspecific proliferation of CD5+ cells [2]. In addition, depending on the bovine lymphocyte antigens genotype and polymorphism in the promoter region of tumor necrosis factor-alpha gene, the infection may progress to B-cell lymphoma [3–6], which is usually found in the lymph nodes, heart, kidney, liver, uterus, eyes, and udder [7]. The presence of lymphoma affects animal health and productivity; however, even cows with PL or at the asymptomatic stage may have reduced milk production and a weakened immune response to challenging pathogens [8].

The spread of BLV in animals requires the transfer of infected cells to non-infected animals. This might occur naturally during pregnancy, from the dam to the fetus, or after birth, by calves ingesting colostrum and/or milk from infected cows [9–11]. Management practices such as ear tagging and dehorning, using contaminated needles for vaccination or tuberculosis diagnosis, and using the same gloves for transrectal uterine palpation [9] further contribute to BLV dissemination. In addition, BLV transmission by hematophagous insects should not be discharged because BVL is already detected in parts of the mouths of insects [12]. BLV integrates its DNA into the host genome upon infection, causing a lifelong infection [13] accompanied by an immunoglobulin G (IgG)-based humoral immune response [14]. The diagnosis of EBL is relatively simple and can be performed by detecting anti-BLV antibodies or BLV DNA in blood or milk samples; however, because the clinical signs of infection are not readily evident, the diagnosis of EBL is usually neglected. Therefore, the virus spread unnoticed throughout the herds. Nonetheless, the control and eradication of EBL can be accomplished through early testing, strict management practices, and the removal of infected calves from the herd [15]. Vaccines to prevent BLV infection are not commercially available and given the nature of retroviral infections, their use might be controversial. Recently, however, an experimental genetically modified vaccine contributed to reduced BLV spread and the surge of clinical symptoms in vaccinated animals [16]; although effective in herds with a high incidence of infection, its use might prevent serological differentiation of BLV-infected from noninfected but vaccinated animals.

Nonetheless, preventing the early transmission of BLV to calves in herds with low EBL incidence is a valuable strategy for eradicating infection. In this scenario, newborn calves that tested positive for BLV DNA or anti-BLV antibodies before suckling should be immediately removed from their herds, and non-infected calves should receive colostrum and milk from seronegative cows. However, the high cost of serological and molecular BLV detection in blood samples and the need for laboratories with expertise in molecular BLV detection might pose impediments to such procedures. Thus, simpler on-farm methods should be evaluated to reduce early BLV transmission to calves [17]. Heat treatment or freezing and thawing [18] of colostrum and milk from BLV-seropositive cows abrogated infectivity, as measured by intraperitoneal inoculation of sheep.

However, data on the infection rates of calves fed frozen or heat-treated colostrum from BLV-seropositive cows are scarce, and this hypothesis deserves further investigation. In this study, we investigated the vertical transmission of BLV and whether pasteurization of colostrum and milk can effectively reduce BLV transmission in suckling calves.

## Materials and Methods

### Ethical approval

The use of animals in this study was approved by the Ethics Committee for the Use of Animals of the Universidade de Passo Fundo (Protocol # 014/2021).

### Study period and location

The study was conducted from May to August 2022 at the University of Passo Fundo, Rio Grande do Sul state, Brazil.

### Study design

Ninety Holstein animals (45 pregnant dams and 45 newborn calves) were used in this study. All cows were tested for antibodies against BLV and divided into seropositive (n = 27) and seronegative (n = 18) cows. At birth, newborn calves were allocated to one of four groups based on the BLV-infected status of the cow (seronegative or seropositive) and treatment of the colostrum (pasteurized or unpasteurized), as follows: (1) Calves from BLV seropositive cows fed pasteurized colostrum (n = 11), (2) calves from seronegative cows fed pasteurized colostrum (n = 9), (3) calves from seropositive cows fed unpasteurized colostrum (n = 16), and (4) calves from seronegative cows fed unpasteurized colostrum (n = 9). All calves were fed colostrum (15% of body weight) during the first 24 h of life, followed by 3 L of milk twice a day for up to 30 days.

### Animal sampling and testing

Blood samples (10 mL) were collected in vacutainer tubes with or without anticoagulants and were used to detect anti-BLV antibodies and BLV DNA. Cows were sampled during early gestation (between 60 and 90 days) to determine their serological status and the experimental group (seropositive or seronegative). At birth, the cows’ colostrum was mechanically obtained; an aliquot was used for DNA extraction, and the remaining was used to feed the calves and, when necessary, was heat-treated for 60 min at 60°C and then cooled down to 32°C; then, the colostrum temperature was raised to 38°C before feeding the calves. Heat treatment was performed using an automated pasteurization apparatus (Dairy Tech Inc., model DT10G, USA).

Blood samples were collected from all calves before sucking (day 0) to investigate the possibility of *in utero* infection by BLV. Additional blood samples were collected 24 h after birth, after colostrum ingestion (day 1), and again on days 7 and 30 after birth. Blood samples were used to detect antibodies to BLV by an in-house enzyme-linked immunosorbent assay (ELISA) assay [19], and samples from days 0 and 30, which were positive for anti-BLV antibodies, were also used to extract DNA to detect BLV DNA and to confirm either *in utero* infection or BLV transmission by colostrum or milk.

### Serological and molecular detection of BLV

Antibodies against BLV were detected in cows using a commercially available ELISA kit (Ingezim BLV Compac 2.0, Spain) and in calves using an *in-house* ELISA kit, as described by Andreolla *et al*. [19].

BLV DNA was detected in blood, colostrum, and milk samples by nested polymerase chain reaction (nPCR), as described by Schwingel *et al*. [20], using a pair of primers designed to amplify *Tax* gene. DNA was extracted from blood using a PureLink Genomic DNA Mini Kit (Invitrogen, Brazil), according to the manufacturer’s instructions. The extraction of DNA from colostrum samples was performed as follows: 10 mL of colostrum was initially centrifuged (600× *g*, 10 min, 4°C) to remove the fat layer; then, the sample was centrifuged again (4000× *g*, 20 min, 4°C) and the colostrum was carefully removed; then, the bottom of the tube was washed with 1 mL of sterile phosphate-buffered saline (pH 7,2) to collect the pelleted cells. From this, 200 μL was used for DNA extraction using the same protocol as that used to extract DNA from blood. DNA was extracted from the milk samples obtained from the tank similarly. The amount of DNA in the samples was measured by spectrophotometry (NanoDrop, Thermo Fisher Scientific, Brazil), and the concentration was adjusted to 10–20 ng/μL. After nPCR, the samples were analyzed by electrophoresis on a 1.5% gel.

## Results

Blood samples were collected from all calves at birth before feeding with colostrum and assayed for anti-BLV antibodies. Among the 27 calves born to seropositive dams, 2 (#198 and #657) (7.4%) had anti-BLV antibodies on day 0 and were also positive for BLV DNA, as assayed by nPCR (Table-1), indicating the occurrence of transplacental infection. All 27 calves received pasteurized or unpasteurized colostrum from seropositive dams. Blood samples were also collected on days 1, 7, and 30 and tested for anti-BLV antibodies; those samples with anti-BLV antibodies on day 30 were further assayed for the presence of BLV DNA. Excluding two calves infected *in utero*, among the remaining 25 calves born from seropositive cows, 15 (60%) of the remaining 25 calves were seropositive for BLV on day 1. With one exception (calf #663), the calves with anti-BLV antibodies on day 1 were also seropositive by day 30. However, one calf (#146) without antibodies against BLV on day 1 was seropositive for BLV on day 30.

**Table-1:** Detection of antibodies against BLV and BLV DNA in newborn calves born from cows seropositive to BLV. Blood samples were collected at birth before colostrum ingestion (D0) and then at D1 (24 h post-colostrum ingestion), D7, and D30 and analyzed for the presence of anti-BLV antibodies and BLV DNA.

Colostrum and milk treatment	Detection of anti-BLV antibodies and BLV DNA in calves born from seropositive cows						

	Calf identification		ELISA				PCR
		
	No.	sex	D0	D1	D7	D30	D30
Pasteurized	146	M	-	-	-	+	+
	182	M	-	-	-	-	-
	184	M	-	+	+	+	-
	194	M	-	+	+	+	-
	196	M	-	+	+	+	+
	198	M	+	+	+	+	+
	199	M	-	+	+	+	+
	650	F	-	-	-	-	-
	654	F	-	+	+	+	-
	655	F	-	+	+	+	-
	670	F	-	-	-	-	-
Unpasteurized	100	F	-	-	-	-	-
	147	M	-	-	-	-	+
	180	M	-	-	-	-	+
	186	M	-	-	-	-	-
	188	M	-	-	-	-	-
	189	M	-	+	+	+	-
	192	M	-	+	+	+	+
	200	M	-	+	+	+	+
	656	F	-	+	+	+	-
	657	F	+	+	+	+	+
	658	F	-	+	+	+	-
	661	F	-	+	+	+	+
	663	F	-	+	+	-	-
	664	F	-	+	+	+	-
	666	F	-	+	+	+	+
	678	F	-	-	-	-	-

ELISA=Enzyme-linked immunosorbent assay, BLV=Bovine leukemia virus, PCR=Polymerase chain reaction, M=Male, F=Female

Among the calves born from seronegative cows, as expected, none had antibodies against BLV on day 0; however, three calves (#193, #195, and #197) had anti-BLV antibodies on days 1 and 7, but not on day 30, and a fourth calf (#187) had anti-BLV antibodies only on day 30 (Table-2).

**Table-2:** Detection of antibodies against BLV and BLV DNA in newborn calves born from cows seronegative to BLV. Blood samples were collected at birth before colostrum ingestion (D0) and then at D1 (24 h post-colostrum ingestion), D7, and D30 and analyzed for the presence of anti-BLV antibodies and BLV DNA.

Colostrum and milk treatment	Detection of anti-BLV antibodies and BLV DNA in calves born from seronegative cows						

	Calf identification		ELISA				PCR
		
	n°	Sex	D0	D1	D7	D30	D30
Pasteurized	75	M	-	-	-	-	-
	193	M	-	+	+	-	-
	195	M	-	+	+	-	-
	669	F	-	-	-	-	-
	671	F	-	-	-	-	-
	672	F	-	-	-	-	-
	675	F	-	-	-	-	-
	676	F	-	-	-	-	-
	680	F	-	-	-	-	-
Unpasteurized	181	M	-	-	-	-	-
	183	M	-	-	-	-	-
	185	M	-	-	-	-	-
	187	M	-	-	-	+/-	-
	197	M	-	+	+	-	-
	652	F	-	-	-	-	-
	653	F	-	-	-	-	-
	660	F	-	-	-	-	-
	662	F	-	-	-	-	-

ELISA=Enzyme-linked immunosorbent assay, BLV=Bovine leukemia virus, PCR=Polymerase chain reaction, M=Male, F=Female

All blood samples collected from the calves on day 30 were assayed for BLV DNA using nPCR. None of the calves born to the seronegative dams had BLV DNA in their blood samples. However, among the calves born from seropositive dams, excluding the two calves infected *in utero*, at least 9 of the 25 (36%) calves had BLV DNA in their blood samples on day 30. Among these, two calves (#147 and # 180) were seronegative for BLV on all sampling days (Table-1).

We also investigated whether pasteurization of colostrum and milk affects the rate of BLV transmission to calves. Within the calves from Group 1 born from seropositive cows that received pasteurized colostrum, and except the *in utero*-infected calf, we detected 3/10 (30%) calves (# 146, 196, 199) with BLV DNA on blood samples collected on day 30, suggesting that infection occurred through colostrum or milk. Furthermore, among the calves born from seropositive dams but that received pasteurized colostrum and milk, and except the *in utero*-infected calf, there were at least 6 out of 15 (40%) calves (#147, 180, 192, 200, 661, and 666) that were found to have BLV DNA in their blood samples collected on day 30 (Table-1). The results were analyzed using Fisher’s exact test which showed that on-farm colostrum and milk pasteurization did not affect the rate of BLV transmission to calves (p > 0.05).

## Discussion

This study demonstrated that BLV is caused by *in utero* infection and transmitted to newborn calves through colostrum and milk. Thirty days after birth, the overall prevalence of BVL DNA in blood (40, 7%) was similar to that in adult cows, indicating that most BLV infections occur early in life. Earlier studies failed to demonstrate that BLV could cause transplacental infection of the fetus [10] and suggested that it was insignificant in the epidemiology of EBL. However, with the development of more sensitive immunological and molecular assays, the transmission of BLV from infected dams to fetuses has been demonstrated, accounting for 4%–18% of infections [21, 22]. Although we assayed a limited number of seropositive dams and calves (27 pairs), we found that 7.4% of newborn calves were infected *in utero* as assessed by detecting anti-BLV antibodies and BLV DNA in the calf blood after birth but before colostrum ingestion.

The mechanisms underlying transplacental BLV infection in calves have not yet been studied. However, the rate of transplacental infection may be higher in cows with PL or lymphoma [22]. In addition, immunological and hormonal mechanisms might regulate the viral load during pregnancy and the passage of BLV to the fetus. For instance, the presence of estradiol during pregnancy [23] stimulates the production of prostaglandin 2, which inhibits the immune response mediated by T helper 1 lymphocyte and reduces the production of interferon-gamma (IFN-γ). This physiological immunosuppression, which is necessary for maintaining gestation, might be intensified during stressful situations and could contribute to the progression of BLV infection. Indeed, deficient production of IFN-γ is usually correlated with PL and the development of lymphomas in cows with BLV infection [4]. Therefore, it is reasonable to assume that *in utero* infection of the fetus might be correlated with a high BLV load in maternal blood, the presence of PL and B-cell lymphoma in cows, and the presence of ambient stressors. The complexity of these interactions and their impact on fetal BLV transmission should be investigated in future studies.

The transmission of maternal antibodies to newborn calves is central to their survival and protects against major pathogens during the 1^st^ month of life. In this study, all calves received colostrum from their dams, regardless of colostrum quality, which is usually evaluated at the farm using a Brix refractometer [24]. Here, excluding two calves infected *in utero*, among the 25 calves born from seropositive dams, we found 15 (60%) calves with antibodies to BLV at D1 after being fed colostrum. The absence of anti-BLV antibodies in at least 10 (40%) calves was unexpected; however, it is possible that the concentration of anti-BLV antibodies in the dams’ blood was low and that the transfer of anti-BLV antibodies to the colostrum and the calf was very low. In addition, we tested the cows for the presence of anti-BLV antibodies early in pregnancy, and the blood levels of anti-BLV antibodies in these cows may have declined during the subsequent months, resulting in poor transfer to colostrum. In addition, the ingestion of colostrum by these calves might have been deficient or even retarded, which might have further contributed to the lower translocation of Igs from the calves’ intestinal tract to the blood [24, 25], leading to negative results in the assay performed with blood samples collected after colostrum ingestion. Furthermore, the assay we used in cows detected antibodies against the BLV gp51 surface glycoprotein, whereas in calves, we used an *in-house* ELISA directed at the p24 capsid protein, which may also account for the differences observed. In contrast, one calf was detected with anti-BLV antibodies only on day 30 (calf #146), which is consistent with early infection, for example, during the passage of the calf through the birth canal of a BLV-infected cow [22] or by ingesting BLV-infected milk [21].

Among the calves from seronegative dams, we found three calves (#193, #195, and #197) with anti-BLV antibodies in blood samples collected on days 1 and 7 but not on day 30. Since the dams were tested for the presence of anti-BLV antibodies only at the beginning of gestation, it is possible that the cows became infected later during pregnancy and thus had anti-BLV antibodies in the colostrum that were transferred to the calves but detected only in the 1^st^ week of life. In addition, considering the half-life and turnover of maternal Igs in calf blood, it is possible that on day 30, the amount of anti-BLV antibodies in calf blood was too low or absent to be detected in our assay. Again, in cows, we detected antibodies against BLV gp51 glycoprotein; in calves, we detected antibodies against capsid protein, which should be considered. Unfortunately, there are no data reporting the duration of maternal anti-BLV antibodies in calf blood. However, the half-life of colostral-derived Igs in calf blood ranges from 9.6 to 17.7 days for IgG1 and IgG2, respectively, and up to 28.5 days [24]. Thus, depending on the number of maternal-derived anti-BLV antibodies in the blood, a calf could be considered positive on D7 but negative on D30.

In addition to antibodies, maternal colostrum contains up to 3 × 10^6^ cells/mL, most of which are leukocytes. B lymphocytes, the target of BLV, may account for up to 5% of colostrum leukocytes. Some maternal-derived cells enter the calves’ circulation, reaching peak levels within 24 h after colostrum ingestion [24]. Thus, B cells harboring BLV may be the major source of infection in newborn calves. Therefore, we hypothesized that the disruption of colostrum cells by pasteurization would ablate BLV transmission to calves. In this study, colostrum and milk were pasteurized on the farm, and we found that the rate of BLV DNA detected at D30 in calf blood was similar between calves that received pasteurized colostrum (3/10; 30%) and calves that received unpasteurized colostrum (6/15; 40%). Unfortunately, because of farm restrictions and management procedures, we could not evaluate the calves over a longer period. Here, excluding the two calves infected *in utero*, at D30 we detected BLV DNA in nine out of 25 calves (36%); for these calves, most likely, the source of infection was colostrum or milk, or infection could have occurred during delivery [21]. In one calf (#146), we detected anti-BLV antibodies only on day 30, and in two calves (#147 and #180), we did not detect anti-BLV antibodies at any time point. These results are consistent with a recent infection in which viral replication was detected by sensitive nPCR but not antibody testing. Thus, considering calves with anti-BLV antibodies on day 1 (15 calves) and BLV DNA on day 30 (6 calves), the rate of transmission through colostrum and/or milk was 40% (6/15), which was similar to the overall rate of transmission found herein. None of the calves from the seronegative dams had BLV DNA in their blood samples. Previous studies [14, 15, 21, 26] have indicated that pasteurization effectively abrogates BLV infectivity. However, most studies on this subject used milk spiked with blood from seropositive cows rather than colostrum or milk from seropositive cows. In one study, milk was pasteurized (63°C for 30 min), and the cells were concentrated by centrifugation, suspended in sterile saline, and inoculated intraperitoneally in adult sheep [14] rather than feeding newborn calves. In our study, farmworkers performed all the procedures (milking, pasteurization, and calf feeding) using farm equipment. Farm personnel were instructed to pasteurize colostrum and milk at temperatures not higher than 60°C and no longer than 30°C to prevent curdling. Under these conditions, BLV DNA was detected on day 30 in calves fed pasteurized colostrum or milk. Colostrum is rich in cells and proteins and has a high-fat content; thus, the natural conditions of colostrum may contribute to reducing the efficacy of pasteurization. Considering that BLV inserts its DNA into the B-cell genome and that pasteurization temperature might not have been high enough to rupture the cells, it is possible that these B-cells containing the BLV genome reached the calf’s intestine and were actively transferred to blood circulation [25], leading to productive infection. In contrast to the previous study [25], the amount of colostrum fed to calves during the first 24 h of life was equivalent to 15% of their body weight, a volume much larger than that used to concentrate the cells injected into the peritoneal cavity of sheep.

Furthermore, we might consider the possibility that the instructions given to farmworkers were not always strictly followed. Nonetheless, the experiment was performed on a dairy farm with good facilities and trained personnel; as such, we believe that it represents the conditions found on most dairy farms in our region. In addition, this study aimed to introduce the pasteurization of colostrum and milk into the farm routine to reduce the transmission of BLV and perhaps other pathogens; however, this strategy should now be re-evaluated to identify possible bottlenecks and weaknesses.

At this farm, the attending veterinarian was aware of the major horizontal routes of BLV transmission and used good management practices to prevent horizontal spread of the virus, such as the use of individual needles (application of vaccines and medication), disposable sleeves during reproductive interventions (rectal palpation), and disinfection of dehorning and ear tagging equipment. In this case, the probability of horizontal BLV transmission was reduced. Thus, in herds with good management practices, the eradication of BLV should consider the strategic elimination of BLV-seropositive cows initiated with those with PL, which are most likely the source of the virus to uninfected animals. In addition, testing newborn calves before colostrum ingestion to detect *in utero* infection and feeding calves colostrum and milk from seronegative cows should be central to eradicating BLV in dairy herds.

### Limitations and strengths of the study

This study has some limitations. First, we cannot rule out BLV transmission from other sources, such as fly bites and iatrogenic transmission. Second, the detection of BLV DNA in the blood of calves does not indicate that the virus is infectious and remains infective throughout life. Because BLV isolation is laborious and usually unsuccessful, no attempts were made to isolate BLV from the blood of calves. In contrast, this study indicates that vertical transmission is central to maintaining BLV in dairy cows and that molecular testing of calves in the 1^st^ months of life should be central to any eradication program.

## Conclusion

We found that up to 40% of BLV infections occurred vertically (*in utero* or through colostrum and/or milk ingestion) early in the calves’ lives. Thus, testing calves seropositive for BLV DNA during the 1^st^ months of life should be considered a central approach for eradicating BLV from dairy farms.

## Authors’ Contributions

DLQ and LCK: Conceived and designed the study. DLQ, KP, and VAR: Collected samples, performed laboratory analyses, and organized the database. DLQ and RF: Methodology and wrote the first draft of this manuscript. LCK: Performed validation and data analysis and critical review. All authors have read and approved the final manuscript.
